# The Herbal Supplements NOZEMAT HERB^®^ and NOZEMAT HERB PLUS^®^: An Alternative Therapy for *N. ceranae* Infection and Its Effects on Honey Bee Strength and Production Traits

**DOI:** 10.3390/pathogens10020234

**Published:** 2021-02-19

**Authors:** Rositsa Shumkova, Ralitsa Balkanska, Peter Hristov

**Affiliations:** 1 Research Centre of Stockbreeding and Agriculture, Agricultural Academy, Smolyan 4700, Bulgaria; rositsa6z@abv.bg; 2 Institute of Animal Science, Department Special Branches, Agricultural Academy, Kostinbrod 2230, Bulgaria; r.balkanska@gmail.com; 3 Institute of Biodiversity and Ecosystem Research, Department of Animal Diversity and Resources, Bulgarian Academy of Sciences, Sofia 1113, Bulgaria

**Keywords:** microsporidia, nosemosis control, phytotherapy, 16S rDNA gene

## Abstract

Honey bees (*Apis mellifera* L.) are the most effective pollinators for different crops and wild flowering plants, thus maintaining numerous ecosystems in the world. However, honey bee colonies often suffer from stress or even death due to various pests and diseases. Among the latter, nosemosis is considered to be one of the most common diseases, causing serious damage to beekeeping every year. Here, we present, for the first time, the effects from the application of the herbal supplements NOZEMAT HERB^®^ (NH) and NOZEMAT HERB PLUS^®^ (NHP) for treating *N. ceranae* infection and positively influencing the general development of honey bee colonies. To achieve this, in autumn 2019, 45 colonies were selected based on the presence of *N. ceranae* infections. The treatment was carried out for 11 months (August 2019–June 2020). All colonies were sampled pre- and post-treatment for the presence of *N. ceranae* by means of light microscopy and PCR analysis. The honey bee colonies’ performance and health were evaluated pre- and post-treatment. The obtained results have shown that both supplements have exhibited statistically significant biological activity against *N. ceranae* in infected apiaries. Considerable enhancement in the strength of honey bee colonies and the amount of sealed workers was observed just one month after the application of NH and NHP. Although the mechanisms of action of NH and NHP against *N. ceranae* infection are yet to be completely elucidated, our results suggest a new holistic approach as an alternative therapy to control nosemosis and to improve honey bee colonies’ performance and health.

## 1. Introduction

The European honey bee, *Apis mellifera* (Linnaeus, 1758) is the most effective and globally distributed pollinator not only of a large number of important crops but also of wild flowering plants, some of which play an essential role in maintaining ecosystem services [1,2,3]. Over the past several decades, there has been a significant reduction in bee colonies, especially in some geographical regions (e.g., North America), which has raised great public and societal concern [4,5]. The decline of the honey bee population has the most tangible effect on food sources for human and livestock, disrupting wild plant pollination and diversity, altering ecological interactions and function, decreasing crop yields, reducing the yield of bee products, a large number of which have important medical value, etc. [6,7,8,9]. A honey bee colony may harbour a wide variety of disease agents and pests, bacteria, fungi, honey bee-associated viruses, parasitic mites and even other insects that try to take advantage of the rich resources contained within bee colonies [10,11]. Among them, *N. ceranae* (Fries et al. 1996) has been implicated in inflicting annually heavy losses on beekeeping [12,13]. Until 2017, two microsporidia were known—*N. apis* (Zander, 1909) (causing nosemosis Type A) and *N. ceranae* (causing nosemosis Type C) [14]. *N. ceranae* is specific for the Asiatic honey bee (*Apis cerana*, Fabricius, 1793); however, presumably after 2003, *N. ceranae* has switched its hosts and has begun to infect the European honey bee (*Apis mellifera*) [15,16]. Nowadays, *N. ceranae* has become widespread microsporidia in many regions in the world [17,18]. Based on ultra-structural and molecular investigations, a new *Nosema* species in *Apis mellifera*, namely *N. neumanni* (Chemurot et al. 2017), was described in Uganda in 2017 [19]. The importance of *N. ceranae* spillover is not limited to the honey bee but also to other insect species like the bee-eater *Merops apiaster* [20], the South American native bumblebee, *Bombus brasiliensis* (Lepeletier, 1836) [21,22], stingless bees and social wasps [23], solitary bees [24], the small hive beetle, Aethina tumida (Murray, 1867) [25], etc. Numerous studies have indicated that *N. ceranae* has become a worldwide distributed microsporidian pathogen, including in Central Italy [26,27], Croatia [28], Lithuania [29], etc. In an investigation on the prevalence of *Nosema* spp. in temperate and subtropical regions, pure *N. ceranae* infection and *N. ceranae*/*N. apis* co-infection were detected in apiaries from both regions, while pure *N. apis* infection was exclusively observed in the subtropical region [30].

It was found that *Apis cerana* showed a higher immune response and lower *N. apis* and *N. ceranae* spore loads than *A. mellifera*, suggesting that Asiatic honey bees may be better able to defend themselves against microsporidia infection [31].

The most prominent negative influences on honey bee colonies include: suppression of the honey bee immune system [32], shortening of worker bee lifespan [33], the decline in colony strength and productivity [34], queen supersedure [35], increased winter losses and colony collapse [36]. All these adverse effects of *Nosema* spp. on honey bee colonies require the search and development of effective strategies against these widespread parasites. For more than several decades, bicyclohexylammonium fumagillin (isolated from the fungus *Aspergillus fumigatus*) has been widely used as an anti-*N. ceranae* antibiotic [37]. Recent studies have shown that this antimicrobial agent is becoming less and less effective against *N. ceranae* infection [37,38]. Some researchers have further found that fumagilin is rather toxic and may provoke tumorigenic formations in humans. Moreover, it has negative effects on bee health and even leads to hyperproliferation of *N. ceranae* spores [39,40]. The observed toxic effects of fumagillin require strict measures regarding its use in many countries [41].

Considering the above, it seems essential to develop new, alternative approaches against nosemosis. Until now, there have been several new basic approaches to control nosemosis in honey bees.

### 1.1. Use of Small Molecules

The use of biologically active small molecules represents a promising approach against nosemosis [38]. A large number of organic compounds have been tested for control of nosemosis. These include: porphyrins (Porphyrin: PP(Asp)2 and Porphyrin: TMePyP), inhibitors of the enzyme methionine aminopeptidase type 2 (MetAP2), phenolic acids (formic acid, oxalic acid, etc.), polyphenol compounds (resveratrol and thymol), etc. [42,43,44]. Although they represent a reliable alternative therapy in the combat against nosemosis, a disadvantage of these compounds is that after their use viable spores remain in beehives, combs, and feces [38]. Thus, there is a real danger of infection or re-infection in the treated honey bee colonies.

### 1.2. RNA Interference as a Gene Regulating Expression Approach

Another approach for treating *N. ceranae* infection is associated with the use of RNA interference (RNAi). RNAi represents a biological process in which small RNA molecules (microRNA (miRNA) and small interfering RNA (siRNA)) inhibit gene expression or translation, by degrading targeted messenger RNA (mRNA) molecules via post-transcriptional gene silencing [45]. RNAi is widely used in human medicine and represents a promising new anticancer approach [46]. In beekeeping, RNAi technology has been used to protect honey bees from infection by various pathogens and parasites [47]. In vitro studies have shown that RNAi can be applied successfully against some honey bee-associated viruses and the ectoparasitic mite *Varroa destructor* (Anderson and Trueman, 2000) [48,49].

Using an RNAi strategy to reduce the expression of some honey bee genes (gene silencing) has been one of the key measures against nosemosis [50,51]. An example of this is the upregulation of the mRNA levels of the naked cuticle gene (nkd) in adult bees by means of *N. ceranae* infection provoking a suppressed host immune function [52]. It has been found that the oral application of nkd double-stranded RNA (dsRNA) in *N. ceranae*-infected bees, i.e., silencing the host nkd gene, can activate the immune response, suppress the reproduction of *N. ceranae*, and improve honey bees’ health status [52]. Another similar strategy, but this time with the use of RNAi-based gene silencing on parasitic DNA, is the downregulation of the gene encoding *N. ceranae* polar tube protein 3 (ptp3) through the application of dsRNA that is homologous in gene sequence [50]. The ptp3 is the part of the polar tube structure relevant to host–parasite interaction, contributing to the parasitic invasion [53]. It has been demonstrated that the oral application of a dsRNA corresponding to the sequences of *N. ceranae* ptp3 gene silences the expression of the corresponding ptp3 in *N. ceranae*-infected bees. As a result, *N. ceranae* load reduction, improvement of host physiological status, and extension of lifespan in infected bees have been observed [49]. The application of this therapy has also its drawbacks. One of them is related to the degradation of the dsRNA molecules inside insects’ guts, which is associated with additional costs regarding the protection of the dsRNA molecules from insect gut nucleases [54]. Other limiting factors include: the insects’ gut pH and the related activity of the restricted enzyme (affecting the stability of dsRNA), the amount of the dsRNA molecules when administered orally in target insects, the length of the dsRNA molecules, and the life stage of the insects (larvae, pupae or adults) [54]. To overcome these obstacles, dsRNAs have been incorporated in liposomes or nanoparticles, and then these particles have been delivered to insects through feeding on an artificial diet [55]. Nanoparticles/liposomes stabilize the dsRNA molecules, thus ensuring the greater efficiency of the RNAi process.

The results obtained from the use of RNAi technology have clearly demonstrated the prospects of its applications in anti-nosemosis therapy, but more research is needed in order to be widely implemented in beekeeping practice.

### 1.3. Use of Organic Extracts and Natural Supplements as an Alternative Holistic Strategy

Another approach against nosemosis is the use of organic extracts and natural supplements. The major advantage is their lower toxicity for both bee colonies and the environment, compared to other chemical compounds [47]. Many investigations have shown that various organic and aqueous natural products do not show any toxicity to honey bees and lead to a decrease in both parasite load and mortality rate caused by *N. ceranae* infection [56,57,58]. Natural compounds, mostly flavonoids and polysaccharides contained in a number of medicinal plants, demonstrate anti-microsporidian activity in honey bees and are applied most often as alcoholic extracts, although some studies dispute the role of the biological activity of flavonoids against *N. ceranae* infection [58]. Propolis, a mixture of resins, wax, and pollen from buds and flowers of plants, enriched with enzymes and subjected to lactic acid fermentation in the digestive system of bees, has strong antimicrobial, antiviral, and antifungal properties [59]. These properties provoke the interest of the scientific community in propolis as an anti-microsporidian drug. In this relation, an ethanolic extract of propolis has been tested in different bee species experimentally infected with *N. ceranae* [59,60,61]. The obtained results from these investigations support the hypothesis that propolis represents an effective and safe product to control *N. ceranae*, while it is interesting to note that bees seem not to use it to self-medicate when infected with these microsporidia [61]. Some studies have indicated that honey and pollen from sunflowers (*Helianthus annuus* L.; Asteraceae) may also reduce the microsporidian infection and increase survival rate in honey bees [62,63].

A number of commercial supplements (HiveAlive^TM^, Api-Bioxal^®^ and ApiHerb^®^, “BEEWELL AminoPlus”, Nozevit^®^, BeePro^®^, MegaBee^®^, etc.) have been tested for anti-*N. ceranae* activity as well [64,65,66,67,68]. For instance, administration of HiveAlive^TM^ and ApiHerb^®^ significantly reduces *N. ceranae* spores load [64,65]. Application of the dietary amino acid and vitamin complex called “BEEWELL AminoPlus” decreases *N. ceranae* spore and protects honey bees from immune suppression by upregulating the expression of genes for immune-related peptides (abaecin, apidaecin, hymenoptaecin, defensin and vitellogenin) [66]. The investigation of Nozevit^®^ (a natural product from plant polyphenols) has shown that this commercial phytopharmacological supplement may improve bee health by decreasing colony spore loads [67]. However, DeGrandi-Hoffman et al. [68] have found that bee colonies fed with the commercial protein supplements BeePro^®^ and MegaBee^®^ exhibited higher levels of black queen cell virus and *N. ceranae* incidence and greater queen losses in comparison to bee colonies feeding on natural forage (*Brassica rapa*—rapini).

### 1.4. Probiotics and/or Prebiotics

The negative consequences associated with the use of antibiotics in the treatment against nosemosis are primarily related to the disruption of the host microbiota and, in some cases, the increased susceptibility to *N. ceranae* infection [69,70]. The use of microbial supplements (probiotics or prebiotics) represents another innovative approach not only for maintaining or restoring intestinal microbiota, but also in the combat against nosemosis. In this aspect, endogenous gut bacteria belonging to *Lactobacilliaceae*, *Bifidobacteriaceae*, and *Acetobacteraceae* families have been found to suppress the development of *N. ceranae*, by reducing the spore load [70,71]. Different commercial probiotics strains (Bactocell^®^ and Levucell SB^®^, Lallemand Inc., “Biogen-N”, “Trilac”, “Lakcid”, etc.) have also been tested as an alternative therapy against *N. ceranae* infections in honey bees [72,73,74,75,76]. The obtained results from these investigations have shown endogenous bacterial strains to be as efficient as commercial strains in terms of survival of honey bees infected with *N. ceranae*, without a pronounced antagonistic effect on the parasite development.

However, some studies have indicated that uncontrolled and unbalanced administration of probiotics to honey bees may cause dysbacteriosis and increase pathogen susceptibility [76,77].

Some prebiotics are also used to control *N. ceranae* infection in honey bees. For example, prebiotic mannan-oligosaccharides (MOSs) are easily fermentable by gut microbiota and their accelerated reproduction allows them to compete with pathogenic bacteria for both nutrients and space in the gut. The β-glucans (glucose homopolymers) are known for their immune-modulating impact on different species, including honey bees [78]. The polysaccharide chitosan stimulates the bee immune system, leading to a decrease in the degree of infection with *N. apis* and to increased bee survival [79].

### 1.5. Other Approaches

The zeolite clinoptilolite is well-known for its powerful antioxidant activity as a dietary supplement in bees infected with *N. ceranae*. Using clinoptilolite has resulted in significant therapeutic effects on honey bees that are naturally infected with *N. ceranae*, without affecting bee physiology [80].

Many beekeepers prefer the use of a natural approach for the treatment of nosemosis. This is no accident, as prolonged use of antibiotics will inevitably lead to resistance, pollute the environment and honey bee products, which will consequently affect human health as well.

Inspired by the use of natural compounds for *N. ceranae* treatment, we decided to test two new herbal supplements, NOZEMAT HERB^®^ (NH) and NOZEMAT HERB PLUS^®^ (NHP), and their influence on *N. ceranae* infection and honey bee health and survival. We believe that this study will contribute to establishing the most effective approaches against nosemosis without a negative impact on the physiological state of the bee colonies and the resulting bee products.

## 2. Results

### 2.1. N. ceranae Infection and Spore Counts

The amplification of the part of the *16S rDNA* gene showed the presence of *N. ceranae* in both experimental groups (NH and NHP) and the control group (C) during the two pre-treatment sessions (August 2019 and April 2020) and about two months after the two post-treatment sessions (October 2019 and June 2020). There were no PCR products in the negative controls. The results from the obtained sequences have shown the presence of *N. ceranae* species after standard nucleotide BLAST (BLASTN programs) in the GenBank database.

**Figure 1:**
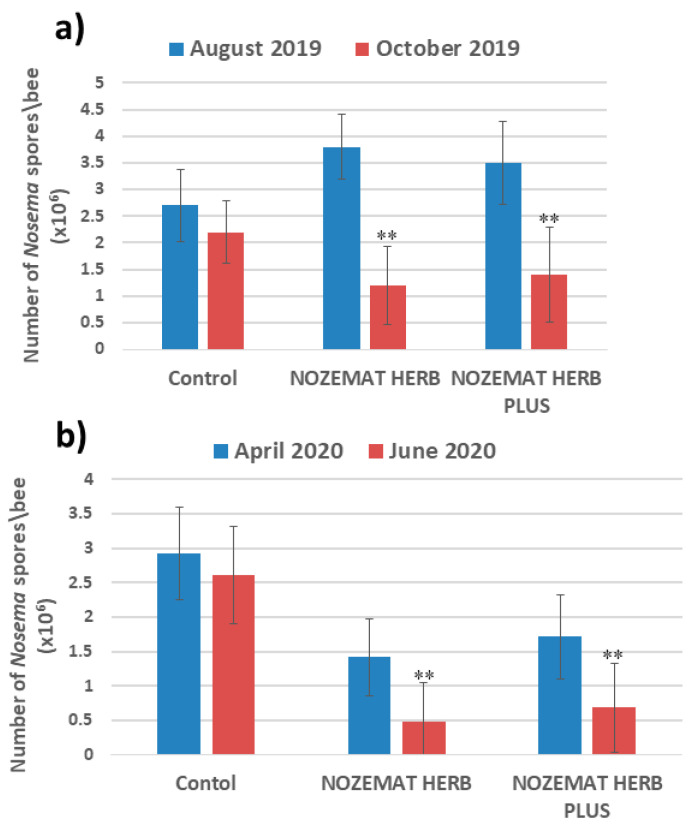
*N. ceranae* spore counts (±SD) pre- and post-treatment with NOZEMAT HERB^®^ and NOZEMAT HERB PLUS^®^ during 2019 (**a**) and 2020 (**b**). The number of *N. ceranae* spores was counted by microscopic examination. Asterisks indicated the level of significance as determined by an analysis of variance followed by Tukey’s multiple comparison test. **: *p* < 0.01 compared to the Nosema spore during pre-treatment.

The numbers of *N. ceranae* spores per bee for the pre- and post-treatment periods each year are shown in Figure 1. Even after the first treatment, a significant reduction in the number of *N. ceranae* spores per bee (*p* < 0.01) was observed (Figure 1a). Compared to the pre-treatment period (August 2019) (NH: 3.8 ± 0.61 × 10^6^; NHP: 3.5 ± 0.78 × 10^6^), in October 2019 the two supplement-treated groups (NH and NHP) showed a significant decrement in *N. ceranae* spore counts: about 68% in the NH group and about 60% in the NHP group (Figure 1a). In contrast to the two experimental groups, the control C group showed a reduction by only 19% of the number of *N. ceranae* spores (pre- treatment 2.7 ± 0.68 × 10^6^; post-treatment 2.2 ± 0.59 × 10^6^). During the next year, at the beginning of the second pre-treatment session (April 2020), in contrast to October 2019, a much more pronounced augmentation of *N. ceranae* spore counts was observed in the control C group—about 33% (ANOVA with Tukey’s HSD post hoc test, *p* = 0.6047; *df* = 8) and less in the experimental groups NH and NHP—18% and 22% (ANOVA with Tukey’s HSD post hoc test, *p* = 0.6104, *df* = 8; *p* = 0.5406, *df* = 8, respectively) (Figure 1b). At the end of the second experimental period in June 2020, compared to the pre-treatment period (April 2020), the NH and NHP groups treated with herbal supplements showed a significant reduction in *N. ceranae* spores (*p* < 0.01)—about 67% (0.47 ± 0.58 × 10^6^) and 60% (0.68 ± 0.64 × 10^6^), respectively (Figure 1b). The reduction in the number of *N. ceranae* spores in the control C group at the end of the second experimental period (June 2020) was only 11% (2.61 ± 0.72 × 10^6^).

The treatment with the herbal supplements NH and NHP showed a significant reduction in the *N. ceranae* spore levels compared to the control C group (post-hoc Tukey HSD Test: *F*  =  24.199, *p* = 0.0004; *F* =  18.919, *p* = 0.0009, respectively).

### 2.2. Effect of Herbal Supplements on Honey Bee Strength and Production Traits

#### 2.2.1. Strength of the Honey Bee Colonies (Estimated Based on Mass)

**Figure 2:**
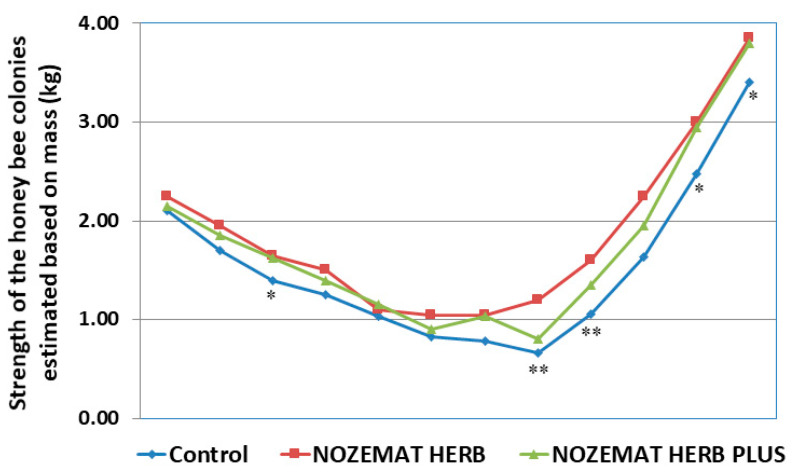
Average (±SD) values in colony strength in honey bee colonies treated with NOZEMAT HERB^®^ and NOZEMAT HERB PLUS^®^ and in an untreated (control) group during 2019 and 2020. Asterisks indicated the level of significance as determined by an analysis of variance followed by Tukey’s multiple comparison test. *: *p* < 0.05, **: *p* < 0.01 compared to the untreated control group.

The strength of the bee colonies was evaluated based on colony mass 12 times during the first pre-treatment (August 2019) and after that 4 times at 12-day intervals post-treatment until September 2020. After the winter period October 2019–February 2020, the same investigations were performed before the second pre-treatment (March 2020) and after that 4 times at 12-day intervals post-treatment until June 2020 (Figure 2). The first difference between the experimental groups (NH and NHP) and the control C group was observed about 25 days after the first treatment (at the end of August 2019). Then, the mass of the bee colonies in the NH and NHP groups was estimated to be 1.65 ± 0.05 kg and 1.63 ± 0.12 kg, respectively, while in the control C group it was 1.25 ± 0.10 kg (*F* =  6.818, *df* = 6, *p* = 0.040; *F*  =  6.943, *df* = 6; *p* = 0.038, respectively). As the autumn period approached, there was a gradual decrease in the studied indicator of bee colony strength in both the control and the two experimental groups, reaching almost full equalization at the end of September(Figure 2). During the second pre-treatment (9 April 2020) the average mass values indicating the strength of the honey bee colonies were significantly higher in the experimental groups than in the control group (ANOVA with Tukey’s HSD post hoc test, *F*  =  26.727, *df* = 8, *p* = 0.002; *F*  =  30.857, *df* = 8, *p* = 0.001, respectively) (Figure 2). The most significant difference was observed in the NHP group, where the mass exceeded 1.2 times that of the control group. This is an indicator of the successful wintering of honey bee colonies and a good start for spring development.

Similar to the first treatment in the previous year (August 2019), the first significant difference between the control and the experimental groups was observed around the 10th day after the second treatment (9 April 2020). Then, the mass (and, accordingly, the strength) of the colonies from the NH group was 1.8 times higher than that of the control C group (*F*  =  31.176, *df* = 6, *p* = 0.001). The difference between the NHP and the control C was about 0.150 kg bees (*F*  =  32.666, *df* = 6, *p* = 0.001). About two months after the second treatment, the difference between the two experimental groups and the control group was about 0.450 kg bees. The mass (and, accordingly, the strength) of the bee colonies of the NH group (1.60 ± 0.07 kg) and NHP group (1.35 ± 0.10 kg) was, respectively, about 1.5 and 1.2 times higher than that of the control group (1.06 ± 0.09 kg) (*F*  =  19.091, *df* = 8, *p* = 0.002; *F*  =  16.90, *df* = 8, *p* = 0.003, respectively). These significant differences persisted during the next measurement period (middle of May 2020), after which there was a less pronounced advantage of the experimental groups over the control group until the last reporting period (June 2020) (Figure 2).

#### 2.2.2. Sealed Worker Brood Area within the Hives

**Figure 3:**
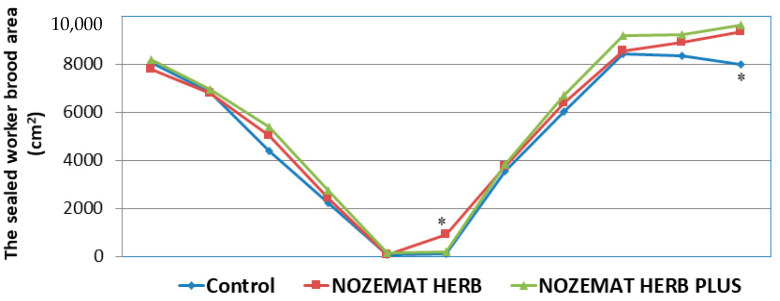
Average (±SD) worker sealed brood area (cm^2^) in honey bee colonies treated with NOZEMAT HERB^®^ and NOZEMAT HERB PLUS^®^ and in an untreated (control) group during 2019 and 2020. Asterisks indicated the level of significance as determined by an analysis of variance followed by Tukey’s multiple comparison test. *: *p* < 0.05 compared to the untreated control group.

A lot of factors determine the queen’s egg-laying rate: age, genotypes, colony size, colony nutrition, etc. It is difficult to quantify the performance of queens relative to workers in the field, and there are not many laboratory assays on queen performance. For this reason, the obtained results on sealed worker brood areas within the hives may not be completely comprehensive, yet this is a parameter that needs to be considered. In contrast to the achieved results regarding the strength of the honey bee colonies, the parameter of sealed worker brood area showed fewer differences after the treatment of the honey bee colonies with herbal supplements (Figure 3). After the first application of NH and NHP (August 2019), the sealed worker brood area gradually decreased both in the experimental (NH and NHP) and in the control C group, which is a biologically determined process in the late autumn period (September 2019). After the winter period and the second pre-treatment (April 2020), the sealed worker brood area in the hives increased; however, only in the NH group (925 ± 29.3 cm^2^) there were statistically significant differences, compared to the NHP (200 ± 31.7 cm^2^) and the control C group (120 ± 26.9 cm^2^) (*F*  =  6.241, *df* = 6, *p* = 0.037; *F*  =  0.303, *df* = 6, *p* = 0.610, respectively) (Figure 3). The second statistically significant difference between the experimental groups and the control group was observed after the fifth month following the second treatment (June 2020). Then, the NH and NHP groups had a significantly larger sealed worker brood area, compared to the control C group (ANOVA with Tukey’s HSD post hoc test, *df* = 8, *F*  =  9.424, *p* = 0.018; *F*  =  13.256, *df* = 8, *p* = 0.008, respectively).

#### 2.2.3. Amount of Capped Honey in the Beehives

**Figure 4:**
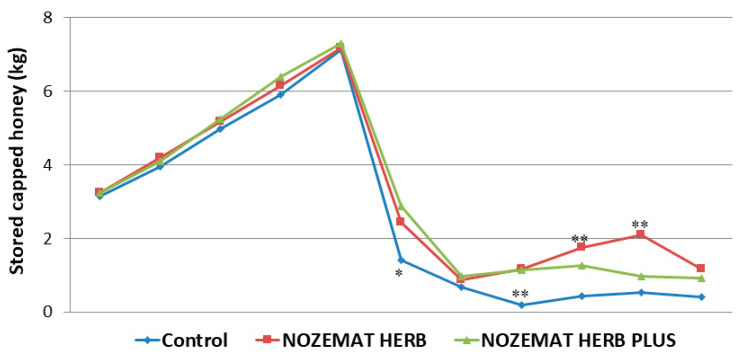
Average (±SD) amount of stored capped honey in honey bee colonies treated with NOZEMAT HERB^®^ and NOZEMAT HERB PLUS^®^ and in an untreated (control) group during 2019 and 2020. Asterisks indicated the level of significance as determined by an analysis of variance followed by Tukey’s multiple comparison test. *: *p* < 0.05, **: *p* < 0.01 compared to the untreated control group.

The results regarding the amount of capped honey in bee combs are presented in Figure 4. As this indicator is directly related to the strength of honey bee colonies (Figure 1), no significant difference was observed between the experimental groups and the control group in the period after the first treatment (August 2019) until the approach of the autumn (September 2019). During the first spring survey in 2020, there were more significant differences in the values of this indicator. About two months after the second treatment (May 2020), there were significant differences between the experimental groups and the control group regarding the amount of capped honey in the beehives. The experimental groups NH (1.18 ± 0.21 kg) and NHP (1.16 ± 0.10 kg) exceeded with about 490% the control C group (0.20 ± 0.13 kg) (*F*  =  77.0395, *df* = 6, *p* = 0.001; *F*  =  148.725, *df* = 6, *p* = 0.001, respectively). During the next two measurement periods (in the middle and at the end of May 2020) this trend persisted, as higher values were observed in the experimental NH group, compared to the control C group (*F*  =  30.015, *df* = 8, *p* = 0.001; *F*  =  62.157, *df* = 8, *p* = 0.001, respectively). During both these periods, the amount of capped honey in the experimental NH group exceeded nearly 4 times this in the control C group. A similar trend was observed between the NHP group and the control C group but with a less pronounced level of significance (Figure 4).

#### 2.2.4. Stored Pollen Area within the Hives

**Figure 5:**
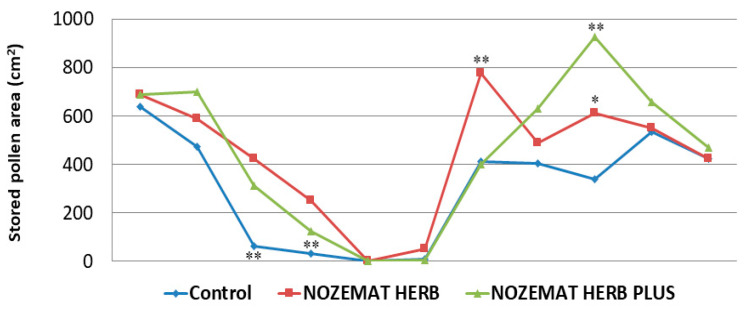
Average (±SD) stored pollen area (cm^2^) in honey bee colonies treated with NOZEMAT HERB^®^ and NOZEMAT HERB PLUS^®^ and in an untreated (control) group during 2019 and 2020. Asterisks indicated the level of significance as determined by an analysis of variance followed by Tukey’s multiple comparison test. *: *p* < 0.05, **: *p* < 0.01, compared to the untreated control group.

The stored pollen area is an indicator of the amount of collected pollen in beehives (a quantity that depends on environmental conditions, the needs of the colonies and the number of bees in the colonies). The stored pollen area was evaluated through direct surface measurements of the comb (cm^2^), and the results are presented in Figure 5. Although the strength of honey bee colonies decreased in all groups, a significant difference was observed between the experimental groups and the control group about 25 days after the first treatment (9 August 2019). Then, the stored pollen area in the experimental groups NH (425 ± 30.8 cm^2^) and NHP (313 ± 31.3 cm^2^) exceeded approximately 7 and 5 times, respectively, that in the control C group (63 ± 32.3 cm^2^) (*F*  =  53.680, *df* = 8, *p* = 0.001, *p* < 0.01; *F*  =  15.491, *df* = 8, *p* = 0.001, *p* < 0.01, respectively). Significantly higher values of this indicator in the two experimental groups compared to the control were observed during the next measurement (the beginning of September 2019) (Figure 5). Then, statistically significant differences were observed between the experimental groups NH and NHP and the control C group (*F*  =  53.172, *df* = 8, *p* = 0.003, *p* < 0.01; *F*  =  23.835, *df* = 8, *p* = 0.002, *p* < 0.01, respectively). During the next year (2020), the most significant difference between the experimental groups and the control group was observed about two months after the second treatment (15 May 2020). In terms of stored pollen area, the NH group surpassed by nearly 80% the control C group (ANOVA with Tukey’s HSD post hoc test, *F*  =  5.631, *df* = 8, *p* = 0.049, *p* < 0.05), while the NHP group exceeded by 170% the control C group (ANOVA with Tukey’s HSD post hoc test, *F*  =  7.966, *df* = 8, *p* = 0.025, *p* < 0.05) (Figure 5).

## 3. Discussion

About 15–20 years after the detection of *N. ceranae* in the Eastern honey bee (*Apis cerana*), these microsporidia became a globally distributed pathogen [81,82,83,84]. Currently, *N. ceranae* is considered one of the main causes of severe annual losses in beekeeping worldwide [85]. For this reason, various strategies have been developed for the control of nosemosis type C caused by *N. ceranae*. It is of crucial importance that the applied approaches are not toxic to honey bees and also to humans (through residues remaining in honey bee products), and are not polluting the environment.

One of the allopathic therapies related to the fight against nosemosis is the use of ecologic phytotherapy [86,87]. It is a suitable remedy against nosemosis due to the low toxicity to bees and the safety of the environment and human health.

A number of studies have focused on the impact of some herbal extracts on the regulation of the expression of certain genes in honey bees in order to reduce the damage caused by *N. ceranae*. For example, it has been shown that plant extracts or decoction from *Andrographis paniculate* promote Wnt and JNK pathways by upregulating the expression of certain genes (including armadillo, basket, frizzled and groucho) in intestinal cells [57]. These results have demonstrated that this Chinese herb can provide protection from *N. ceranae* infection under laboratory conditions, suggesting that it can be used in apiculture to control *N. ceranae*. Another study has revealed that *Eleutherococcus senticosus* extract contains eleutherosides (eleutheroside B + E), which have an impact on the honey bee immune system [88]. These eleutherosides increase phenoloxidase (PO, a major defense enzyme in many invertebrates) in hemolymph and inhibit the development of fungal spores. The piperine (an alkaloid in the roots of the Piperaceae family) and curcumin (a natural phenol produced by *Curcuma longa*) are known as natural supplements which increase the activity of the antioxidant system in honey bees [89,90]. They promote the activities of antioxidant enzymes such as superoxide dismutase, peroxidase, catalase and glutathione S-transferase, which reduces oxidative stress [91]. Moreover, it is observed that these herbal supplements exhibit hydroxyl radical scavenger action in honey bees, suppressing the destructive effects of the free radicals and reactive oxygen species [92]. Because *N. ceranae* is able to induce oxidative stress in bees [93], piperine and curcumin are potential candidates regarding antinosemosis therapy.

The present study is the first report of the in vivo application of two plant extracts, NOZEMAT HERB^®^ (NH) and NOZEMAT HERB PLUS^®^ (NHP), against *N. ceranae* and their impact on honey bee colonies. These products are patented herbal supplements for honey bees. NH contains herbal extracts, vegetable glycerin, water, citric acid, and preservative—potassium sorbate. NHP contains additional herbal extract as well as vitamin C (ascorbic acid). The exact quantitative composition of these two supplements is patent-protected and thereby not disclosed in this paper.

After a sequence analysis of the part of the 16S rDNA gene, we obtained a fragment with 219 bp. Then, we performed a BLAST analysis in the GenBank genetic sequence database to identify regions of local similarity between the available sequences for the *N. ceranae* 16S rDNA gene. We found the highest homology of our sequences (Acc. no. MG657260) with *N. ceranae* isolates from Argentina (Acc. no. KX024757) and Lithuania (Acc. no. JQ639314). Besides the conventional PCR analysis for detection of *N. ceranae* based on sequence analysis of 16S rDNA gene, a recently developed new method not only provides rapid and reliable detection of the presence of these microsporidia but also allows the quantification of *N. ceranae* (qPCR assay) based on the highly-conserved protein coding gene Hsp70 [94].

In both years of our study (2019–2020), a significant reduction of *N. ceranae* spore loads was observed after NH and NHP administration, compared to the pre-treatment level. Among the benefits from these two supplements is their positive effect on the intestinal microflora of honey bees. Numerous studies have indicated that microbial communities have an essential role in the resistance to pests and pathogens, environmental toxins and pesticide exposure [77,95,96,97]. As for the *N. caranae* infection, there is evidence that these microsporidia disrupt the microbiota, causing dysbiosis, which may have consequences on bee development and immune suppression [97,98,99]. As an intestinal infection, *N. ceranae* causes significant changes in the composition of honey bee gut microfauna, and the plant extracts that we use contain biologically active substances that have a beneficial effect on gut bacterial communities. In fact, both products (NH and NHP) contain basic polyphenols—flavonoids and phenolic acids—as biologically active compounds. According to some authors, the antimicrobial activity of plant extracts is not due to a single biologically active substance (flavonoids vs phenolic acid), but rather to the totality of all, with potentially synergistic effects [59,100]. Phenolic compounds extracted from *Artemisia dubia* and *Aster scaber* have shown a clear anti-nosemosis effect, which is a promising strategy for controlling nosemosis [101,102]. As far as flavonoids are concerned, the studies carried out so far have not identified a single representative that can be effective on its own against nosemosis [59,65]. Unfortunately, the mechanism of action of both flavonoids and phenolic compounds against *N. ceranae* has not been elucidated yet. The latter would certainly help to speed up the process against these microsporidia.

A lot of research has been carried out on the application of plant extracts supplementation against various pathogens in bee colonies, e.g., *V. destructor* and honey bee-associated viruses, *N. ceranae*, American foulbrood (*Paenibacillus larvae*) [66,87,103,104,105], or pests, e.g., the greater wax moth *Galleria mellonella* [106]. In addition to the effect of plant extracts, the potential beneficial effects of herbal extracts on honey bee colonies have been tracked as well. Certain researchers purposefully reflect on the impact of various plant extracts on honey bee performance [107,108,109,110,111].

Our study aimed to evaluate the impact of the herbal supplements NOZEMAT HERB^®^ and NOZEMAT HERB PLUS^®^—on the one hand, as an alternative therapy against nosemosis, and on the other hand, considering the influence of the two products on honey bee colony strength and honey production and pollen collection for two consecutive years (2019 and 2020).

We found a significant reduction of *N. ceranae* spores load after administration of NH and NHP, compared to the pre-treatment period both in 2019 and in 2020. In contrast to the experimental groups, the control C group exhibited a less clear decrease of *N. ceranae* spores levels (Figure 1). These data are consistent with other studies in which a significant decrease of *N. ceranae* spores load was established after the application of other commercial food supplements, such as HiveAlive™ [64] and ApiHerb [65]. A similar effect was found in Chinese herbal extracts (in particular *Andrographis paniculata*), when applied as a decoction to treat *N. ceranae* infections in *A. mellifera* [55]. These results indicate that plant extracts represent a potent alternative therapy against nosemosis.

During the first year (2019) of our research, the administration of the two herbal supplements did not reveal significant differences between the experimental NH and NHP groups compared to the control C group with regard to colony strength (Figure 2). These results are an indicator of a decrease in the strength of bee colonies as autumn approaches. These data support the findings of Charistos et al. [64], who also did not observe differences between the groups in terms of colony strength after the administration of HiveAlive™ food supplement during the autumn. The first informative review in the spring of the following year (2020) showed a significant increase in colony strength in both experimental groups in contrast to the control group, which is an indicator of the more successful overwintering of the treated bee colonies. This trend continued after the second treatment (April 2019) until the beginning of the autumn period. These data support previous investigations about the positive influence of herbal supplements such as HiveAlive™, *Laurus nobilis* L. and *Agaricus brasiliensis* extracts on honey bee colony strength [64,104,107].

Similar to the indicator of bee colony strength, the sealed worker brood area did not show significant differences in all the groups after the first herbal supplement administration (Figure 3). The first informative review in the spring of the following year (2020) showed a larger sealed worker brood area in the NH group, but as a whole, this indicator was not affected by the applied plant extracts.

The amount of capped honey was affected most significantly after the second treatment of the bee colonies in April 2020 (Figure 4). After that, the amount of capped honey was significantly higher in the two experimental NH and NHP groups compared to the control C group. This indicator maintained significantly higher levels in the two experimental groups throughout the summer, which is a prerequisite for successful wintering of the bee colonies and reducing the costs associated with additional feeding during the winter.

It is interesting to note that the stored pollen area was significantly affected after the first treatment (August 2019) despite the presence of sparse flowering vegetation (Figure 5). After the second treatment the following year, a larger stored pollen area was observed in the experimental groups, and it seemed that the NHP group had an advantage over the NH and the control C group in terms of this indicator.

Although the findings of the present study clearly demonstrate the benefits from applying the two plant extracts for the reduction of *N. ceranae* spore counts as well as for honey bee performance in general, these encouraging results call for further research in order to clarify the impact of NOZEMAT HERB^®^ and NOZEMAT HERB PLUS^®^ on *N. ceranae* spore loads as well as on honey bees.

## 4. Materials and Methods

### 4.1. Ethics Statement

All experimental procedures were reviewed and approved by the Animal Research Ethics Committee of the Bulgarian Food Safety Agency (BFSA), (Ar. 154 from of the Law on Veterinary Activity) in accordance with the European Union Directive 86/609.

### 4.2. Experimental Design

The study was conducted during the period of 11 months (5 August 2019–8 June 2020) at the Experimental Apiary of the Research Center of Stockbreeding and Agriculture—Smolyan, Bulgaria (41°35′7.01″ N, 24°41′30.98″ E). The apiary is located in Smolyan municipality—the Perelik-Prespa part of the Western Rhodope Mountains. The apiary consists of 60 colonies of *Apis mellifera rodopica* (local ecotype of *A. m. macedonica*) housed in Langstroth Rut hives. All hives have exposure to the same environment and the same forage conditions.

For the purposes of our investigation, 45 of those colonies were selected based on the presence of *N. ceranae* infection, which was detected in the 25 forager bees sampled from each colony both microscopically and with PCR analysis. Forager bees can be distinguished from house bees based on appearance (with less, darker hairs in the chest area), presence of visible pollen load on their legs, and location in the hive (mainly in the part of the combs occupied by food supplies). Considering the presence of *N. ceranae* infection, the 45 honey bee colonies were divided into three groups—two experimental groups NH (*n* = 15) and NHP (*n* = 15), and a control group C (*n* = 15). The bee colonies were equalized in terms of bee colony strength, sealed worker brood area, amount of honey and stored pollen area.

The treatment of the bee colonies with herbal supplements was carried out once in the autumn (5 August 2019) and once in the spring (9 April 2020). The bee colonies in the experimental groups were treated 4 times at 7-day intervals with NOZEMAT HERB^®^ and NOZEMAT HERB PLUS^®^ (Extract Pharma, Sofia, Bulgaria) at a dose of 10 mL of the product dissolved in 100 mL of sugar syrup (1:1, w/w), according to the manufacturer’s instructions (Extract Pharma Ltd., Sofia, Bulgaria). The solution was sprayed with a syringe onto the bee combs in each experimental hive. The bee colonies of the control C group were sprayed only with sugar syrup (1:1, w/w), at the same dose as the experimental groups.

Before each pre-treatment (August 2019 and April 2020) and about two months post-treatment (October 2019 and June 2020), 20 forager honey bees were sampled from each colony from the three investigated groups for microscopic (stored at 4 °C) and PCR analysis (stored at −20 °C) for *N. ceranae* examination.

### 4.3. Microscopic Detection of N. ceranae

The bees were sent to the National Reference Laboratory for Bee Diseases at the National Diagnostic Science and Research Veterinary Medical Institute (Sofia, Bulgaria) in a cooler bag. The abdomens of 20 forager honey bees from each colony of the experimental groups and the control group were macerated in 3 mL of distilled water, and the pellets were filtered and centrifuged for 10 min at 1000× *g*. A 100 μL aliquot was placed on a microscope slide and covered with a coverslip. *N. ceranae* spores were counted at ×400 magnification. Positive samples were recounted for an accurate spore count using a Neubauer hemocytometer on (0.1 μL volume). One *N. ceranae* spore observed in the entire hemocytometer’s grid (25 × 16 = 400 small squares) was equal to an average of 1500 spores per bee. The reported information was the number of spores per bee [112].

### 4.4. DNA Extraction, PCR Amplification and Sequencing

After light microscopy, a total of 150 spore samples from each investigated colony were investigated by a PCR analysis in the experimental groups and the control group in the pre-treatment period. For each of the suspensions of isolated *N. ceranae* spores, an aliquot of 50 μL was transferred to a new tube and centrifuged at 15,000× *g* for 10 min. The total DNA was isolated from the obtained supernatant by using a GeneMATRIX Tissue DNA purification kit (Cat. no. E3550, EURx Ltd., Gdansk, Poland) as per the manufacturer’s instruction. Briefly, the pellet was resuspended in a buffer Lyse T, and 20 μL of Proteinase K were added and incubated overnight at 56 °C with shaking. The quality and quantity of the isolated DNA were checked by 1% agarose gel electrophoresis and then visualized under UV trans-illuminator gel documentation systems after staining with SimpliSafe™ (cat. no. E4600; EURx Ltd., Gdansk, Poland). The isolated DNA was stored at −20 °C before analysis.

Considering that *N. ceranae* is becoming a globally distributed pathogen, we decided to perform molecular detection on these microsporidia.

The small-subunit (SSU) rRNA (*16S rDNA*) gene was chosen for molecular detection of *N. ceranae*, using the primers 218MITOC—FOR (5′-CGGCGACGATGTGATATGAAAATATTAA-3′) and 218MITOC—REV (5′-CCCGGTCATTCTCAAACAAAAAACCG-3′) designed by Martín-Hernández et al. [113]. Negative controls were included in all PCR experiments. As a positive control, cytochrome c oxidase subunit 1 (*coI*) gene fragment of *Apis mellifera* was used in all the studied samples. The sequence of primers used for positive control was CoI2-F (5′-CCTGATATAGCATTTCCTCG-3′) and CoI2-R (5′-TGTGAATGATCTAAAGGTGG-3′) designed on the basis of the complete mitochondrial genome of *A. m. ligustica* (Acc. no. L06178) [114].

All PCR reactions were performed with 10 ng template DNA in a final volume of 50 μL (NZYTaq II 2 × Colourless Master Mix, cat. no. MB354; NZYTech, Lda.—Genes and Enzymes, Lisbon, Portugal). The PCR conditions were as follows: initial denaturation at 94 °C for 5 min; 30 cycles of denaturation at 94 °C for 30 s, primers hybridization at 50 °C for 30 s, elongation at 72 °C for 1 min, and final elongation at 72 °C for 10 min. The successfully amplified products for *N. ceranae* were purified with a GeneMATRIX PCR/DNA Clean-Up Purification Kit (cat. no. E3520; EURx Ltd., Gdansk, Poland) and sequenced in both directions using a PlateSeq kit (Eurofins Genomics Ebersberg, Germany).

### 4.5. Evaluation of Honey Bee Strength, Sealed Worker Brood and Food Supplies

During the investigation period, 12 measurements were performed in all investigated groups (experimental and control) to determine the strength of the colonies (based on mass) and 11 measurements to determine the sealed worker brood area, the amount of honey and the stored pollen area. These measurements were performed once pre-treatment (August 2019) and four times post-treatment, at 12-day intervals, until the end of September (2019). After the winter period, the same measurements were performed once before the second pre-treatment (April 2020) and four times post-treatment, at 12-day intervals, until the beginning of June (2020).

The following parameters characterizing the development of the bee colonies were determined:

1. Strength of the bee colony based on its mass (kg)—the mass is calculated based on the number of frames occupied by bees, considering that one frame in a Langstroth–Rut hive contains approximately 200 g of bees [115].
2. Sealed worker brood area—a measuring frame with the size of the squares 5 × 5 cm was used. In 1 cm^2^ there were 4 worker cells in the bee comb. The area of 25 cm^2^ corresponded to 100 worker cells [115].
3. Amount of honey in the beehives—a measuring frame with 5 × 5 cm squares was used to measure the capped honey in the bee combs. Eight squares of the measuring frame corresponded to 0.350 kg honey [115].
4. Stored pollen area in the beehives—the amount of the collected pollen was evaluated through direct surface measurements of the comb (cm^2^) [115].

### 4.6. Statistical Analysis

The dependent variables studied and normalized by linear transformation were: number of *N. ceranae* spores, strength (mass) of honey bee colonies, sealed worker brood area, amount of honey, and stored pollen area. These variables in the investigated groups were compared using F-statistic, a one-way ANOVA analysis of variance (IBM SPSS Statistics 23.0 for Windows) with a post hoc Tukey HSD test for multiple comparisons with Bonferroni and Holm correction.

The obtained sequences (219 bp the part of *16S rRNA* gene) were deposited in the GenBank database under accession numbers MG657260.

## 5. Conclusions

This is the first study to evaluate in vivo the effects of NOZEMAT HERB^®^ and NOZEMAT HERB PLUS^®^ on *N. ceranae* spore loads, honey bee strength and production traits for two consecutive years. Both herbal supplements increase the strength of the bee colonies, with NOZEMAT HERB^®^ showing a stronger impact. The sealed worker brood area does not seem to be significantly affected by the treatment. However, both supplements seriously increase the amount of capped honey, and this effect is maintained throughout the year. The supplements also increase the stored pollen area throughout the year, with NOZEMAT HERB PLUS^®^ having a greater effect.

Both herbal supplements can be successfully used as an alternative therapy against nosemosis. Furthermore, they are not toxic to bees and are easily ingested by the latter. As plant extracts, they are completely safe for humans, animals, and the environment. Therefore, they can be fed as natural food supplements all year round in combination with sugar syrup or as honey bread for winter feeding.

## Data Availability

The data presented in this study are available on request from the corresponding author.
